# Efficacy of Digital Anesthesia: Comparison of Two Techniques

**Published:** 2017-09

**Authors:** Muhammad Ahmad

**Affiliations:** Department of Aesthetic Plastic Surgery, Islamabad Private Hospital, Islamabad, Pakistan

**Keywords:** Digital anesthesia, Subcutaneous ring block, Transmetacarpal technique

## Abstract

**BACKGROUND:**

Digital nerve block is commonly performed by care providers in medical fields. This study compares the blocks in terms of effectiveness of anesthesia and pain.

**METHODS:**

Patients were divided into two groups. First group underwent digital block whereas 2^nd^ group had transmetacarpal digital block. The subcutaneous ring block was performed by two injections of 3 ml of 2% lignocaine in a 3 ml syringe with a 26G needle at the level of phalangeal/palmer crease. One prick was performed on either side of the finger base extending on dorsal and volar aspects of the digit. The transmetacarpal block received lignocaine identically at dorsal aspect of metacarpo-phalangeal joint. 1.5ml of the solution was injected in dorsal and 1.5ml in palmer side on either side of the finger. When sensation of needle was felt, 1ml of the solution was injected. Then the needle was withdrawn injecting another 1ml and finally the last 1ml was injected close to the dorsal skin. The pain prick was recorded after 30 seconds.

**RESULTS:**

The mean time to complete abolition of sensation was 9.1 minutes in group I and 9.0 minutes in group II. The mean duration of anaesthesia was 202 minutes in group I and 206.8 minutes in group II. The mean pain scale was 5.67 (range=4–7) in group I and 4.2 (range=3–7) in group II.

**CONCLUSION:**

Subcutaneous ring block and transmetacarpal techniques are good in digital anesthesia and involve the administration of the local anaesthetic through two injections.

## INTRODUCTION

Digital nerve block is one of the most commonly performed blocks by care providers in several medical fields.^1^ Various studies have been conducted demonstrating the efficacy and safety of local anaesthesia as a digital block.^2^^-^^5^ There are various methods of performing this procedure including *(i).* the traditional subcutaneous block with one or two punctures, *(ii).* circumferential subcutaneous ring block at the finger base, *(iii) *the transmetacarpal block via two dorsal punctures, *(iv)* transthecal block and, (v) SIMPLE block (Single subcutaneous Injection in Medline of Phalanx with Lignocaine and Epinephrine).^6^^-^^8^ Dorsal skin is said to be less painful to needle punctures than the volar glabrous skin. The classic two injections dorsal approach was originally described by Braun and Harris.^9^ The transmetacarpal and the ring blocks have not been compared in any local study. Both of these require two injections on either side. The objective of the study was to compare the blocks in terms of effectiveness of anesthesia and pain.

## MATERIALS AND METHODS

This study was conducted in a private setup from January 2013 to December 2013. The study goal was to compare time to abolition of distal sensations and pain from the procedure. The patients were divided into two groups. First group comprised of the patients undergoing digital block whereas 2^nd^ group consisted of the patients having transmetacarpal digital block. All the patients underwent surgery distal to the proximal phalanx crease. The patients were randomly selected for either group. Informed consent was obtained and only adult patients >18 years of age were included. Any patient having history of allergic reaction to Lignocaine, neurological disease, peripheral vascular disease, diabetes mellitus was excluded from the study. 

**Fig. 1:**
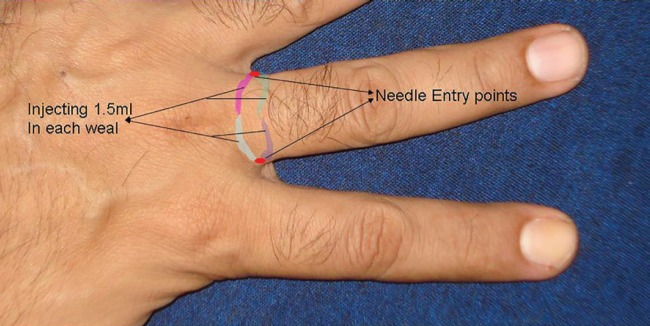
Ring Block

All the blocks were performed by the single investigator and all the testing was also performed by the same investigator. The same dose and concentration of local anaesthetic was used for each technique. The subcutaneous ring block was performed by two injections of 3 ml of 2% lignocaine in a 3 ml syringe with a 26G needle at the level of phalangeal/palmer crease (Figure 1). One prick was performed on either side of the finger base extending on dorsal and volar aspects of the digit. The transmetacarpal block was performed by injecting 3 ml of 2% lignocaine through the dorsal aspect at the level of metacarpo-phalangeal joint. 1.5ml of the solution was injected in dorsal and 1.5ml in palmer side on either side of the finger. 

**Fig. 2:**
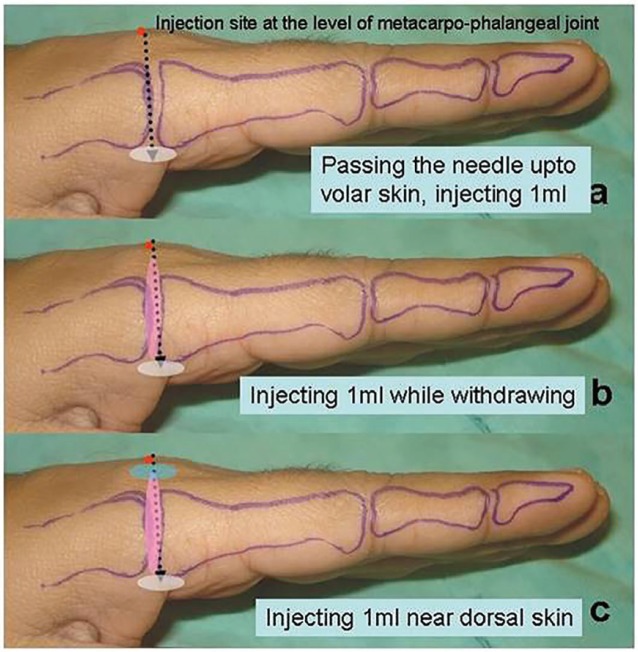
Transmetacarpal block

The tip of the surgeon’s finger was placed on palmer side. The needle was pushed through and through but not out of the palmer skin. The feel of the needle was felt and about 1ml of the solution was injected. Then the needle was withdrawn injecting another 1ml in the way and finally last 1ml was injected close to the dorsal skin. The same procedure was repeated on the other side (Figure 2). Time was noted while administering the injections. The sensations to pain prick was noted after 30 seconds. The patient was asked about the pain according to the Pain Scale (0–10) (Table 1).

**Table 1:** Pain scale.

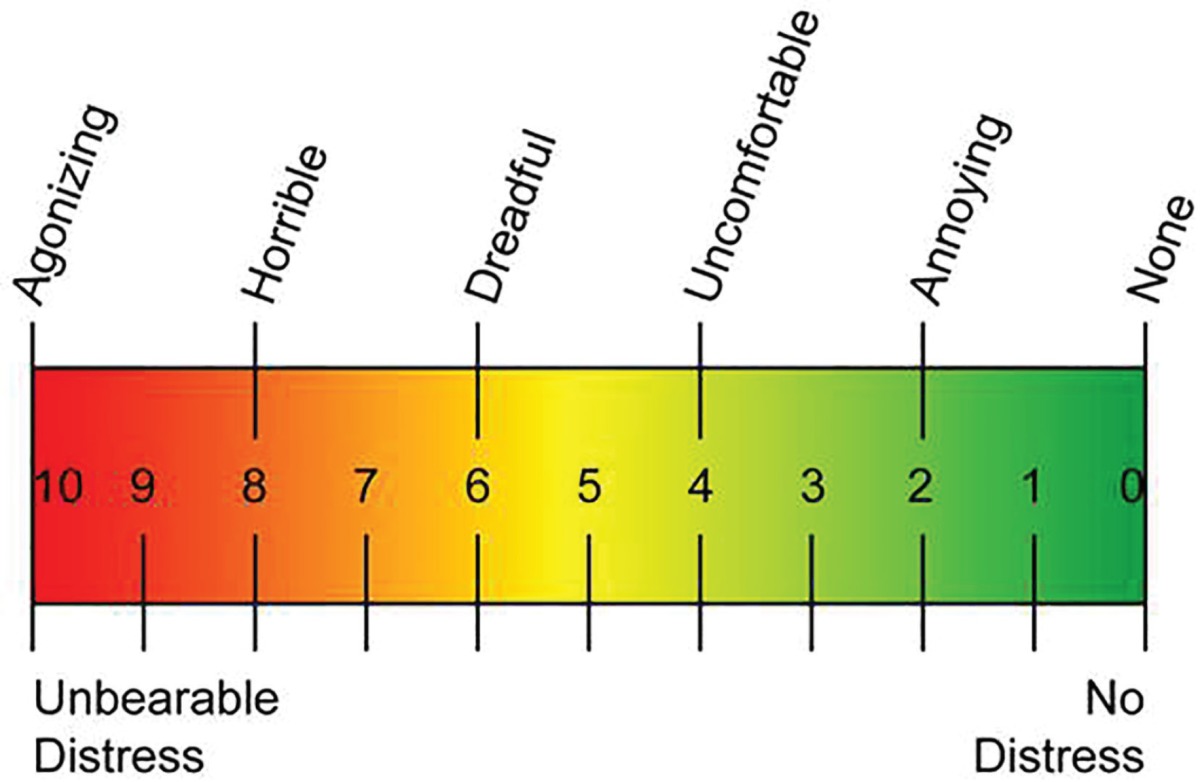

## RESULTS

Fifteen patients were included in either group. Mean age of the patient was 29.2 years in group I (ring block) and 27.4 years in group II (transmetacarpal block). Male to female ratio was 2:1 and 1.5:1 in group I and II respectively. The mean time to complete abolition of sensation was 9.1 minutes in group I and 9.0 minutes in group II (range: 7-11 and 7-11 respectively). The mean duration of anaesthesia was 202 minutes in group I and 206.8 minutes in group II. The mean pain scale was 5.67 (range=4–7) in group I. The mean pain scale in group II was 4.2 (range=3–7).

## DISCUSSION

The ideal method of digital block anaesthesia would have a quick onset, painless and result in complete anaesthesia of the whole digit including volar as well as dorsal digital skin. The level of anaesthesia should provide total abolition of pain and light touch sensations. Various techniques of digital block have been described.^6^^-^^8^ Digital subcutaneous ring block involves the injection on either side of the base of finger extending the needle to dorsal and volar side. Transmetacarpal block involves the injection from the dorsal skin at the base of metacarpo-phalangeal joint.^6^^-^^8^

In the present study, we compared the two techniques, i.e., transmetacarpal block and digital nerve block. Both these techniques require two injections (one on either side) to abolish the sensations. The transmetacarpal block involved the puncture of dorsal skin advancing to the palmer side, where feel of the needle was felt by the surgeon’s other hand, thereby avoiding skin puncture. The mean time between the two techniques was insignificant. 

The mean pain score in ring block was 5.67 as compared to 4.2 in transmetacarpal block. This is in contrast to the observation noted by Knoop *et al.* which revealed no significant pain rating,^4^ whereas the time for abolition of sensation was 6.35 minutes vs 9.1 minutes and 2.82 minutes vs 9.0 minutes. The reason for this obvious difference in time may be due to the fact that we checked the pain sensation on the injured/affected finger whereas in the study by Knoop *et al.*, the sensation was checked on the opposite side of the injured finger.^4^

Failure rate to achieve complete anaesthesia was 23% and 3% in subcutaneous block and transmetacarpal block respectively in the study by Knoop *et al*. whereas no case of failure to achieve anesthesia was seen in the present study.^4^ The reason is that Knoop *et al.* used different doses of lidocaine for each method. But similar doses and volumes were used in the present study in both the groups (i.e., 3ml of 2% lidocaine without adrenaline).^4^ Various other studies have been conducted to compare the results of transthecal and transmetacarpal blocks,^2^^,^^3^^,^^10^ and the results of transthecal and subcutaneous blocks.^11^ No study has been found comparing the results of subcutaneous ring block and transmetacarpal block in the local literature.

Various theoretical drawbacks are associated with transmetacarpal block. Firstly, the adjacent finger is also anesthetized. Secondly, there is a theoretical chance of having a direct injury to a nerve or vessel because, needle passes very close to the neurovascular bundle but none was found in the present study. Pulling back the plunger of the syringe before injecting confirms the needle is not in the vessel. The comparison of the present study is shown in Table 2 with some of the other studies already conducted. Subcutaneous ring block and transmetacarpal techniques both are good in achieving digital anaesthesia. Both of them involve the administration of the local anesthetic through two injections.

**Table 2:** Comparison of various studies

**Study **	**Journal**	**Year**	**Techniques**	**Sample size**	**Pain scale**	**Mean Score**	**Mean time to anesthesia**	**Failed anaesthesia**	***Conclusion***
Ahmad et al. (Pakistan)	Current study	2010	Ring block & Transmetacarpal block	15 each	Analogue pain scale 0–10	5.67 & 4.2	9.1 & 9.0	NIL	*Both effective*
Hill *et al*.^3^ (USA)	Ann Emerg Med	1999	Transthecal & Traditional Digital Block	31 volunteers	Visual analogue	Mean analog score 1.7 & 1.4	188 & 152		*Both equal*
Knoop *et al*.^4^ (USA)	Ann Emerg Med	1994	Digital block & metacarpal block	30 patients	Non segmental visual analogue	Mean analogue score 2.53 & 3.38	2.82 & 6.35 minutes	3% & 23%	*Digital block more effective*
Bashir *et al*.^10^ (Pakistan)	J Coll Physicians Surg Pak	2008	Dorsal digital block & Volar block	30 patients	Pain scale score 0–10	5.27+1.05 & 4.27+0.87	_______	________	*Volar block more effective*
Willaim *et al*.^8^ (Canada)	Plast Reconstr Surg	2006	Doral block & Volar block	27 volunteers	______	______	_______	________	*No significant difference*
Hung* et al.*^1^* (USA)*	J Hand Surg (B)	2005	Subcutaneous, Metacarpal & Transthecal	50 volunteers	Analogue pain score	________	______	______	*Subcutaneous block preferred*
Brutus* et al.*^13^*(Belgium)*	Chir Main	2002	Transthecal, Subcutaneous & Combination of both	30 patients	Visual analogue pain scale	83.3%	______	16.7%	*Subcutaneous preferred *
Low* et al.*^11^* (USA)*	J Hand Surg (Am)	1997	Transthecal & Subcutaneous	20 volunteers	______	______	______	_______	*Subcutaneous preferred*
Cannon* et al.*^12^* (UK)*	*Emerg Med J*	*2010*	*Subcutaneous & Digital nerve block*	*37 & 39*	NIL (clinician satisfaction)	89% & 82%	_______	_______	Subcutaneous preferred

## CONFLICT OF INTEREST

The authors declare no conflict of interest.
